# Physiological basis for isoxadifen-ethyl induction of nicosulfuron detoxification in maize hybrids

**DOI:** 10.1371/journal.pone.0173502

**Published:** 2017-03-07

**Authors:** Lanlan Sun, Renhai Wu, Wangcang Su, Zenggui Gao, Chuantao Lu

**Affiliations:** 1 Department of Plant Protection, Shenyang Agricultural University, Shenyang, China; 2 Institute of Plant Protection, Henan Academy of Agricultural Sciences, Zhengzhou, China; Clemson University, UNITED STATES

## Abstract

Isoxadifen-ethyl can effectively alleviate nicosulfuron injury in the maize. However, the effects of safener isoxadifen-ethyl on detoxifying enzymes in maize is unknown. The individual and combined effects of the sulfonylurea herbicide nicosulfuron and the safener isoxadifen-ethyl on the growth and selected physiological processes of maize were evaluated. Bioassays showed that the EC_50_ values of nicosulfuron and nicosulfuron plus isoxadifen-ethyl for maize cultivar Zhengdan958 were 18.87 and 249.28 mg kg^-1^, respectively, and were 24.8 and 275.51 mg kg^-1^, respectively, for Zhenghuangnuo No. 2 cultivar. Evaluations of the target enzyme of acetolactate synthase showed that the I_50_ values of nicosulfuron and nicosulfuron plus isoxadifen-ethyl for the ALS of Zhengdan958 were 15.46 and 28.56 μmol L^-1^, respectively, and were 0.57 and 2.17 μmol L^-1^, respectively, for the acetolactate synthase of Zhenghuangnuo No. 2. The safener isoxadifen-ethyl significantly enhanced tolerance of maize to nicosulfuron. The enhanced tolerance of maize to nicosulfuron in the presence of the safener, coupled with the enhanced injury observed in the presence of piperonyl butoxide, 1-aminobenzotriazole, and malathion, suggested cytochrome P450 monooxygenases may be involved in metabolism of nicosulfuron. We proposed that isoxadifen-ethyl increases plant metabolism of nicosulfuron through non-P450-catalyzed routes or through P450 monooxygenases not inhibited by piperonyl butoxide, 1-aminobenzotriazole, and malathion. Isoxadifen-ethyl, at a rate of 33 mg kg^-1^, completely reversed the effects of all doses (37.5–300 mg kg^-1^) of nicosulfuron on both of the maize cultivars. When the two compounds were given simultaneously, isoxadifen-ethyl enhanced activity of glutathione S-transferases (GSTs) and acetolactate synthase activity in maize. The free acid 4,5-dihydro-5,5-diphenyl-1,2-oxazole-3-carboxylic was equally effective at inducing GSTs as the parent ester and appeared to be the active safener. GST induction in the maize Zhenghuangnuo No. 2 was faster than in Zhengdan 958.

## Introduction

Nicosulfuron (NS) is a sulfonylurea herbicide that provides postemergence control of broadleaf weeds and sedge weeds in maize. It has become the most frequently used herbicide in China because of its high herbicidal activity at low application rates [1]. However, some cultivars of maize may be injured by its application. Recent studies have shown that some corn hybrids present different levels of tolerance to nicosulfuron [2–6]. Li et al. [5] reported that the maize hybrid Zhengdan958 had moderate resistance. To date, very little information is available about the levels of tolerance in Zhenghuangnuo No. 2 to nicosulfuron.

The level of injury from nicosulfuron is substantially affected by alleles at a single locus, presumably the Nsf1/Ben1 locus, or closely linked loci on the short arm of chromosome 5 [7,8]. Crop injury occurs when genotypes and other factors affect herbicide action. Studies on selectivity highlight the need to observe some factors, such as the hybrid used, seasonal period of nitrogen fertilizer application, the phenological stage of the crop at the time of application, meteorological conditions, and improper usage and dosage. If any of these factors are neglected, crop selectivity to nicosulfuron may be reduced, leading to the appearance of phytotoxic effects in the corn plants [9–11].

Injury to heterozygous cultivars may be reduced if herbicides are formulated with safeners [8]. Safeners have been shown to enhance the activity of P450 monooxygenases [12]. Differential sensitivity in field corn to P450-metabolized herbicides has been associated with different rates of metabolism [13,14]. Previously, Bunting et al. [14] found that isoxadifen-ethyl increased foramsulfuron absorption, translocation, metabolism, and crop tolerance of a hybrid injured by foramsulfuron alone. Several new and emerging herbicide products formulated with P450-enhancing crop safeners, such as isoxadifen-ethyl, may reduce injury to heterozygous- and homozygous-tolerant hybrids [8].

All safeners primarily work by enhancing the degradation of the herbicides to inactive metabolites in the crop [15,16]. The speed of these transformations is the main reason for crop selectivity and weed control [17]. The major mechanism by which currently developed safeners protect crops from herbicidal injury is through enhancement of herbicide detoxification. Safeners enhance activity of degradative enzymes, such as cytochrome P-450 monooxygenases, glutathione S-transferases (GSTs), and UDP-dependent glycosyltrasferases.

Safeners, such as isoxadifen-ethyl and mefenpyrdiethyl, can enhance sulfonylurea herbicide tolerance in cereal crops by effectively inducing cellular xenobiotic detoxification mechanisms [18]. The safener 1,8-naphthalic anhydride can reduce the phytotoxicity of chlorimuron-ethyl to maize by inducing acetolactate synthase (ALS) and GST activity [19].

A strong correlation was observed between the efficacy of a safener and its ability to induce GST activity, suggesting that herbicide tolerance in safener-treated plants is a result of the induced ability to detoxify the herbicide via GSH conjugation [20]. Likewise, species-specific differences in herbicide tolerance can also be explained by differences in GST activity and the capacity for herbicide detoxification by glutathionylation. The importance of GSTs for the inactivation of herbicides notwithstanding, many more enzymes are involved in the various phases of xenobiotic detoxification, all of which appear to be induced by safeners [20–22].

Maize tolerance to nicosulfuron is attributed to rapid conversion of the parent herbicide to the nonphytotoxic 5-pyrimidine-OH metabolite. Metabolism of nicosulfuron in corn occurs by hydroxylation of the pyrimidine ring, and hydroxylation of nicosulfuron is inhibited by malathion [23]. Since nicosulfuron is hydroxylated by cytochrome P450 monooxygenases (P450), insecticide interference with P450 activity may be the cause of increased herbicide injury. Corn injury from the interaction of organophosphate insecticides with sulfonylurea herbicides has been attributed to reduced herbicide metabolism in insecticide-treated plants [24,25]. The use of herbicide safeners to compensate for reduced sulfonylurea herbicide metabolism in insecticide-treated plants is of interest because several safeners have increased the metabolism of sulfonylurea herbicides [26,27].

Numerous studies have shown information pertaining to the levels of injury to nicosulfuron in sweet corn and some common maize cultivars, and the protection from nicosulfuron injury to sweet corn provided by herbicide safeners. There has been relatively little research examining the differences in the tolerance of maize hybrids Zhengdan 958 and Zhenghuangnuo No. 2 to nicosulfuron and the effects of safener isoxadifen-ethyl on detoxifying enzymes in maize.

The objectives of this study were to: (1) determine the physiological basis for the protective action of isoxadifen-ethyl, and (2) determine if P450 inhibitors affect maize tolerance to nicosulfuron in the presence and absence of isoxadifen-ethyl. Our hypothesis was that the effects of these P450 inhibitors would be offset by isoxadifen-ethyl, which can increase plant metabolism of herbicides through non-P450-catalyzed routes or through P450 monooxygenases not inhibited by ABT, PBO, and malathion. Our second hypothesis was that isoxadifen-ethyl appeared to induce the GST activity involved in the herbicide detoxication.

## Materials and methods

### Plant material and growth conditions

Maize seeds were planted in plastic pots filled with a 2:1:1 mixture of soil, vermiculite, and growing medium. The pots had holes in the bottom to allow them to absorb water from dishes placed under each pot. The pots were then placed in a growth chamber and submitted for the first two days to darkness at 21°C (relative humidity at 80%), and thereafter to day—night conditions (12 h light at 26°C, light intensity of 300 μmol m^-2^ s^-1^, and 12 h of darkness at 21°C). The pots were watered daily. They were treated at 7 d after sowing. Before treatment, the seedlings were thinned to 5 plants per pot. The treatments were applied using methods modified from Schulte and Köcher [28]. The different treatments were applied to the leaf core of maize plants using a micro syringe. The applied volume per plant was 50 μL.

For the whole-plant bioassay, nicosulfuron was evaluated at 4.7, 9.4, 18.75, 37.5, 75, 150, and 300 mg kg^-1^ in combination with or without isoxadifen-ethyl at 33 mg kg^-1^. After treatment, the plants were then returned to the controlled environment. Shoot length and dry weight were evaluated one week after treatment (WAT). For the GSH and GST extraction and analysis, leaves were collected 1, 3, 5, and 7 d after treatments, immediately frozen in liquid nitrogen, and stored at -80°C.

For the influence of the P450 inhibitors on isoxadifen-ethyl activity, nicosulfuron was evaluated at 20 mg kg^-1^; PBO, ABT, and malathion were measured at 10 mg L^-1^; and the herbicide safener isoxadifen-ethyl was assessed at 33 mg kg^-1^. These concentrations were chosen from a previous dose response experiment (data not shown). Maize seedlings were treated with PBO, ABT, malathion, nicosulfuron, isoxadifen-ethyl, PBO or ABT or malathion plus nicosulfuron or isoxadifen-ethyl, and PBO or ABT or malathion plus nicosulfuron plus isoxadifen-ethyl. After treatment, the pots were incubated for 7 d; shoot length and dry weight were measured and recorded.

For the GST induction studies with isoxadifen-ethyl, 7-day-old maize shoots were treated with isoxadifen and the free acid 4,5-dihydro-5,5-diphenyl-1,2-oxazole-3-carboxylic (IEM) at 33 mg kg^-1^ to determine whether the ester group on isoxadifen has any function in its ability to safen maize. Maize shoot was harvested 4h, 8h, 12h, 24h, and 48h after treatment in order to determine how quickly isoxadifen and IEM exert safening. After harvest maize shoot was weighed, frozen in liquid nitrogen and stored at -80°C.

### Determination of ALS activity

ALS was extracted from 7-day-old etiolated maize shoots using methods of enzyme extraction from Fan et al. [29]. The shoots were homogenized in 4 volumes of buffer containing 0.1 mol L^-1^ K_2_HPO_4_, pH7.5, 1 mmol L^-1^ sodium pyruvate, 0.5 mmol L^-1^ MgCl_2_, 0.5 mmol L^-1^ thiamine-pyrophosphate, 10μmol L^-1^ FAD and 10% v/v glycerol. The homogenate was filtered through 8 layers of cheesecloth and centrifuged at 25,000g for 20min under the temperature of 4°C, the supernatant fluid was precipitated by (NH_4_)_2_SO_4_ at about 50% of saturation for 2 h at 0°C. The enzyme pellet after centrifugation at 25 000g for 30 min under the temperature of 4°C. was dissolved in ALS dissolving solutions (pH 7.0, 50 mmol L^-1^ K_2_HPO_4_-KH_2_PO_4_ buffers containing 20 mmol L^-1^ pyruvate, 0.5 mmol L^-1^ MgCl_2_). This solution of crude enzyme was used immediately for ALS specific activity determination.

The procedure of in vitro ALS assay was conducted according to the method of Fan et al. [29]. 0.1 ml aliquot of inhibitor was added to 0.4 ml enzyme and 0.5 ml enzyme reaction solution (pH 7.0, 50 mmol L^-1^ K_2_HPO_4_-KH_2_PO_4_ buffers containing 24 mmol L^-1^ pyruvate, 0.6 mmol L^-1^ MgCl_2_, 1 mmol L^-1^ TPP, and 20 μmol L^-1^ FAD), then heated to 35±1°C for 1 h in the dark, 100μl of 3 mol L^-1^ H_2_SO_4_ was added and incubated at 60±1°C for 15 min to decarboxylate, then, a 0.5 ml 5% aliquot of freshly prepared a-naphthol in 2.5 mol L^-1^ NaOH and 0.5 ml 0.5% creatine was added to the mixture to determine the acetoin by color development at 60±1°C for 15 min. The red color complex was cooled to temperature by water and the absorbance was measured at 525 nm. ALS specific activity was demonstrated as nmol acetoin mg^-1^ protein h^-1^. Protein has been measured by the method of Coomassie blue according to Bradford [30].

### Determination of GSH content and GST activity

For determination of GSH content, a 0.2 g tissue of maize shoot was homogenized in 5% sulfosalicylic acid and centrifuged at 15,000 *g* for 20 min. The supernatant was used for GSH content measurements by adding potassium phosphate buffer (pH = 8.0) and DTNB as the chromogenic agent. Absorbance was determined at 412 nm, whereas GSH content was estimated from a standard curve and reported as μmol GSH g^-1^ Fresh weight [31].

The extraction and GST assay were performed as described by Mimmo et al. [32]. The GST activity was calculated by the extinction coefficient ε = 9.6 mM^-1^ cm^-1^ and expressed in amount of conjugate constituted by GSH and CDNB catalyzed by GST per unit time per mg of enzyme (μmol min^-1^ mg^-1^ protein).

### Statistical analysis

All data presented here are the mean values of two independent experiments with four replicates. Data are presented as mean ± standard deviation (SD). Statistical analyses were performed by analysis of variance using SPSS software. Differences among treatment means were compared using a least significant difference test at the 5% level of significance. The ANOVA (SPSS Version 20.0, SPSS Inc,) showed no significant difference between the two run experiments, and the data were pooled. I_50_ (the herbicide dose required to cause 50% reduction in ALS activity) and EC_50_ (the herbicide dose required to cause a 50% reduction in plant height) were calculated using the following probit model (Eq 1). The analysis was performed using SPSS software.

Y = A + BX (1)

where Y is the probit, A is the intercept, B is the regression coefficient, and X is log_10_(dose) [33].

## Results

### Effect of nicosulfuron and isoxadifen-ethyl on maize seedlings growth

The results obtained from the whole-plant bioassay confirmed that nicosulfuron was a potent inhibitor of plant height of both Zhengdan 958 and Zhenghuangnuo No. 2 maize (Table 1). The EC_50_ values of nicosulfuron for Zhengdan 958 and Zhenghuangnuo No.2 were 18.87 and 24.8 mg kg^-1^, respectively. When the herbicide safener isoxadifen-ethyl was added to nicosulfuron, the EC_50_ values of nicosulfuron for Zhengdan 958 and Zhenghuangnuo No.2 were 249.28 and 275.51 mg kg^-1^, respectively. These results demonstrated that isoxadifen-ethyl safened maize against injury from nicosulfuron.

**Table 1:** EC_50_ and I_50_ values affected by nicosulfuron alone or in combination with isoxadifen-ethyl.

Treatment^a^	EC_50_ (mg kg^-1^)		I_50_ (μmol L^-1^)	
	ZD	ZHN	ZD	ZHN
NS^b^	18.87	24.8	15.46	0.57
NS+IE	249.28	275.51	28.56	2.17

^a^ All nicosulfuron and safener treatments were applied with a non-ionic surfactant (0.1% v/v).

^b^ Abbreviations: EC_50_, effective concentration of nicosulfuron that causes a 50% reduction in plant height; I_50_, the concentration of nicosulfuron that reduced ALS activity by 50% for *in vivo* experiments; ALS, acetolactate synthase; NS, nicosulfuron; IE, isoxadifen-ethyl; ZD, Zhengdan 958; ZHN, Zhenghuangnuo No. 2.

**Fig 1:**
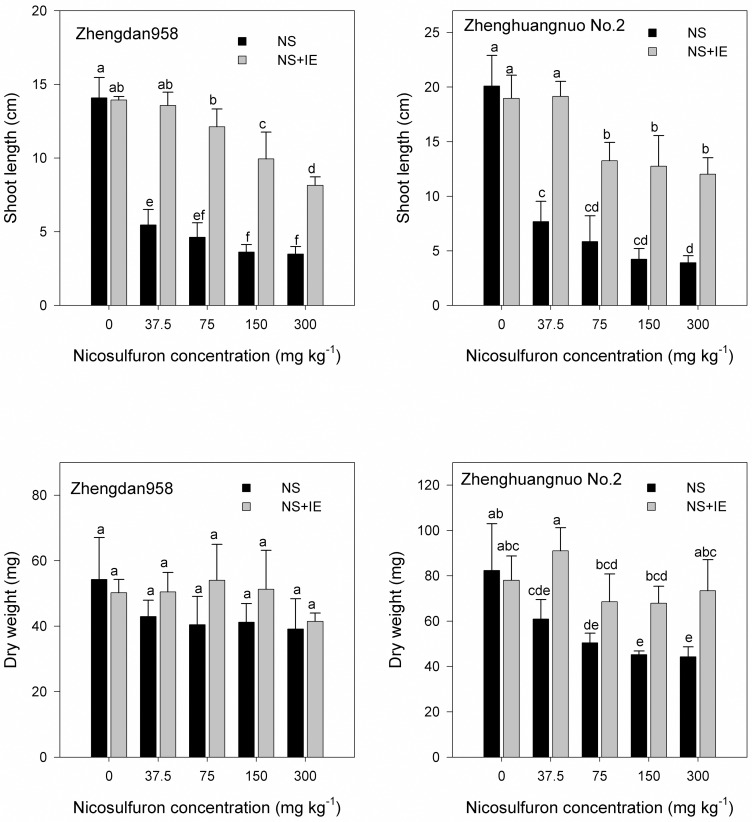
Shoot length and shoot dry weight in maize Zhengdan 958 and Zhenghuangnuo No.2 exposed to nicosulfuron alone or in combination with isoxadifen-ethyl seven days after treatment. The different lowercase letters are significantly different from each other (P < 0.05) among different concentrations of nicosulfuron according to Duncan’s test. NS: nicosulfuron; IE: isoxadifen-ethyl.

Based on the whole-plant bioassay, subsequent studies on the interaction of nicosulfuron with isoxadifen-ethyl were conducted using the herbicide at concentrations of 37.5, 75, 150 and 300 mg kg^-1^. Under these conditions, nicosulfuron exerted a severe shoot growth retardation. Treatment with 37.5, 75, 150 and 300 mg kg^-1^ of nicosulfuron reduced shoot length by 38.8%, 32.9%, 25.7% and 24.8%, respectively, in Zhengdan 958 maize and by 38.2%, 29.1%, 21.1% and 19.5%, respectively, in Zhenghuangnuo No. 2 maize. Dry weight was reduced by 79.1%, 74.5%, 75.9% and 72.1%, respectively, in Zhengdan 958 maize and by 74%, 61.2%, 54.8% and 53.7%, respectively, in Zhenghuangnuo No. 2 maize (Fig 1).

Isoxadifen-ethyl alone did not inhibit growth of the two maize hybrids (Fig 1). Isoxadifen-ethyl mitigated the effects of all concentrations of nicosulfuron on both maize hybrids. There was a significant difference between nicosulfuron alone and nicosulfuron plus isoxadifen-ethyl for both of the maize hybrids. The protection offered by isoxadifen-ethyl on both hybrids of maize was excellent against 37.5 mg kg^-1^ of nicosulfuron. The shoot lengths of Zhengdan 958 and Zhenghuangnuo No. 2 were reduced by 96.2% and 95.4%, respectively, when treated with nicosulfuron plus isoxadifen-ethyl. The protection offered by isoxadifen-ethyl to maize against 75 and 150 mg kg^-1^ of nicosulfuron was partial and slightly better in Zhengdan 958 than in Zhenghuangnuo No. 2 maize (Fig 1). In addition, the protection offered by isoxadifen-ethyl on both maize hybrids against nicosulfuron injury depended on the nicosulfuron concentration.

### *In vitro* ALS specific activity of two maize cultivars as influenced by nicosulfuron

The results of *in vitro* ALS specific activity determination are listed in Table 1. The results indicated that nicosulfuron and nicosulfuron plus isoxadifen-ethyl inhibited ALS activity of maize Zhengdan958 and Zhenghuangnuo No.2. The inhibition of nicosulfuron on the ALS of the two maize hybrids was stronger than that of nicosulfuron plus isoxadifen-ethyl, and the I_50_ values of nicosulfuron to the ALS of Zhengdan958 and Zhenghuangnuo No.2 were about 1.85 and 3.81 times lower than that of nicosulfuron plus isoxadifen-ethyl. There were large differences in inhibitions of the same herbicide between the two maize cultivars. These results differed from the whole-plant bioassay result. Differences in metabolism likely explain this result. Herbicide that was taken up by maize could be transported and metabolized by the whole plant, and other enzymes, such as multifunctional oxidase and GST. Metabolizing herbicide was found in living maize during the whole-plant bioassay, while other enzymes that metabolize herbicide were not found during assessment of ALS specific activity. The results also indicated that Zhenghuangnuo No. 2 would be injured by nicosulfuron more easily than Zhengdan 958. Considering only the sensitivity of the whole plant, the chance of nicosulfuron hurting the two maize cultivars was slim. Metabolism and environment were found to be the most important integrated factors affecting the phototoxicity to maize.

### Effect of nicosulfuron, isoxadifen-ethyl, and cytochrome P450 inhibitor on maize seedling growth

**Fig 2:**
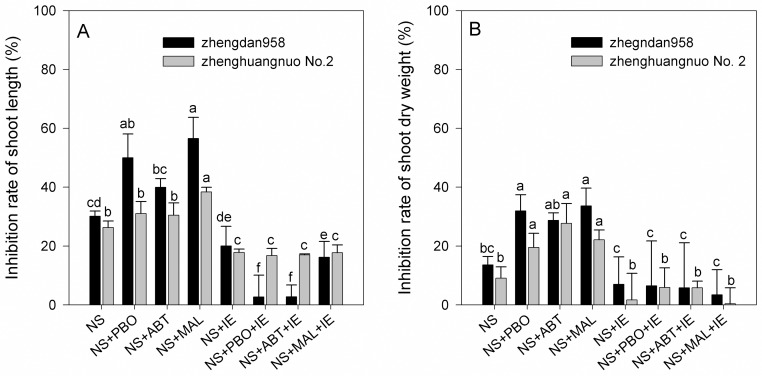
Shoot length (A) and shoot dry weight (B) of maize leaf tissue seven days after treatment. Plants were treated with nicosulfuron (20 mg kg^-1^) alone or in combination with the P450 inhibitors PBO, ABT, or malathion. Maize was also treated with the herbicide safener isoxadifen-ethyl alone or in combination with PBO, ABT, or malathion. A non-herbicide treated control is included for comparison. Columns containing the same letter are not statistically different as determined by Duncan’s test (P<0.05). NS: nicosulfuron; IE: isoxadifen-ethyl; PBO: piperonyl butoxide; ABT: 1-aminobenzotriazole; MAL: malathion.

The responses of maize seedlings treated with nicosulfuron or nicosulfuron + isoxadifen-ethyl, with or without PBO, ABT and malathion are shown in Fig 2.

For Zhengdan 958 cultivar, nicosulfuron alone reduced shoot length and shoot dry weight by 30.1% and 13.6%, respectively, compared to non-treated control plants. Applications of PBO, ABT, or malathion alone did not affect maize growth (data not shown). However, the addition PBO, ABT, or malathion to nicosulfuron noticeably increased the inhibition of maize growth. The shoot length was reduced by 50%, 40%, and 57%, respectively; while shoot dry weight was reduced by 32%, 29%, and 34%, respectively. The addition of isoxadifen-ethyl to nicosulfuron significantly decreased the inhibition of maize.

For Zhenghuangnuo No. 2 cultivar, nicosulfuron-induced inhibition of shoot length increased from 26.3% when applied alone to 31%, 30%, and 38% when applied with PBO, ABT, or malathion, respectively. Inhibition of shoot dry weight increased from 9.1% when applied alone to 19.6%, 27.7%, and 22.1% when applied with PBO, ABT, or malathion, respectively. Compared to nicosulfuron alone, the inhibitions of plant height or biomass treated with nicosulfuron plus isoxadifen-ethyl plus ABT, PBO, or MAL were significantly decreased.

Applications without nicosulfuron, malathion, ABT, PBO, IE, or combinations did not cause any reductions in shoot length or shoot dry weight compared to the non-treated control.

### Effect of nicosulfuron and isoxadifen-ethyl on shoot GSH content

**Fig 3:**
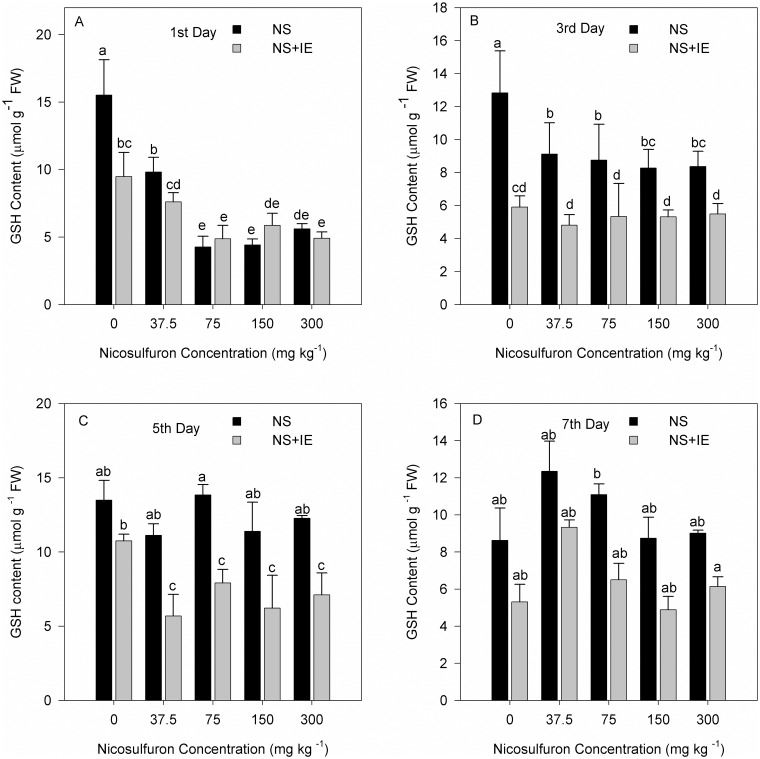
Changes in GSH content in maize Zhengdan 958 exposed to nicosulfuron alone or in combination with isoxadifen-ethyl after treatment. The different lowercase letters are significantly different from each other (P < 0.05) among different concentrations of nicosulfuron according to Duncan’s test. NS: nicosulfuron; IE: isoxadifen-ethyl.

On the first and third days, the GSH content of maize Zhengdan 958 decreased after treatment with nicosulfuron or nicosulfuron plus isoxadifen-ethyl compared to the control. GSH content was found to be higher in maize plants treated with nicosulfuron compared to the nicosulfuron plus isoxadifen-ethyl treatment. It was statistically determined that GSH content decreased on the fifth day in the groups treated with isoxadifen-ethyl (Fig 3).

**Fig 4:**
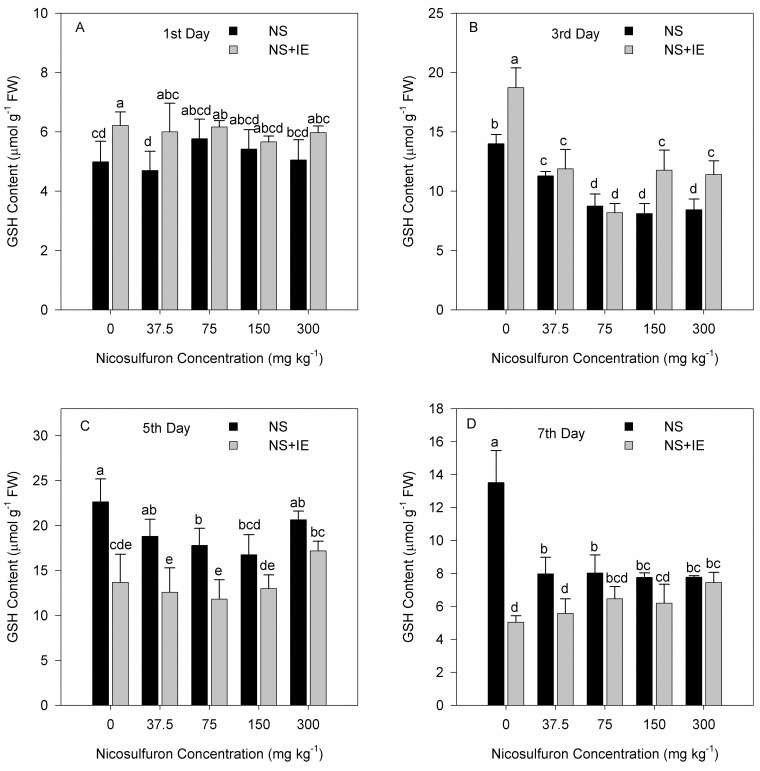
Changes in GSH content in maize Zhenghuangnuo No. 2 exposed to nicosulfuron alone or in combination with isoxadifen-ethyl after treatment. The different lowercase letters are significantly different from each other (P < 0.05) among different concentrations of nicosulfuron, according to Duncan’s test. NS: nicosulfuron; IE: isoxadifen-ethyl.

For Zhenghuangnuo No. 2 cultivar, the GSH content increased significantly in the isoxadifen-ethyl alone treatment on the first and third days. The GSH content decreased significantly after treatment with nicosulfuron alone or in combination with isoxadifen-ethyl. In the nicosulfuron or nicosulfuron plus isoxadifen-ethyl treated plants, the GSH content increased in the nicosulfuron treated groups compared to the nicosulfuron plus isoxadifen-ethyl treated on the fifth and seventh days (Fig 4).

### Effect of nicosulfuron and isoxadifen-ethyl on shoot glutathione S-transferase activity

The GST activity of maize increased after treatment with nicosulfuron alone or in combination with isoxadifen-ethyl.

**Fig 5:**
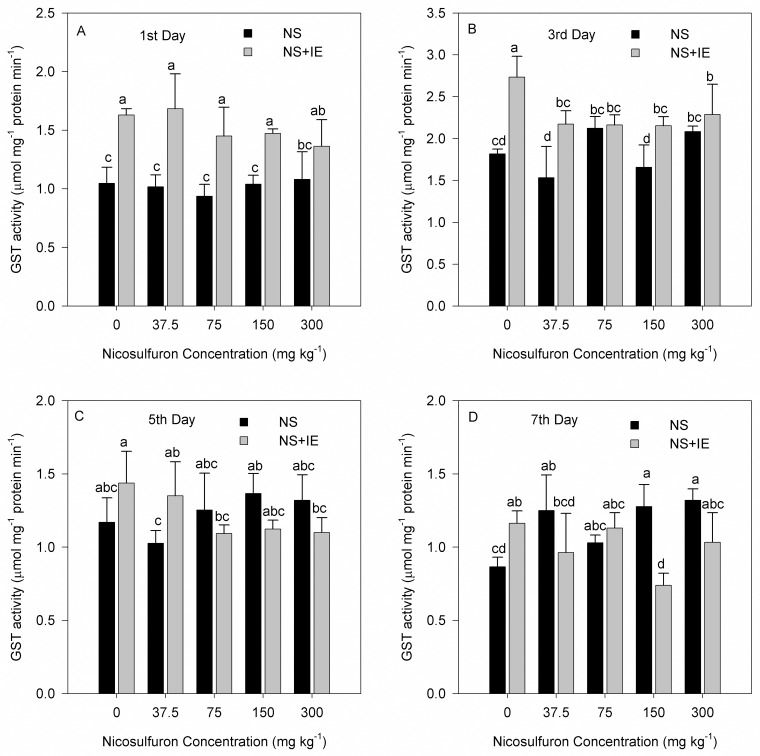
Changes in GST activity in maize Zhengdan 958 exposed to nicosulfuron alone or in combination with isoxadifen-ethyl after treatment. The different lowercase letters are significantly different from each other (P < 0.05) among different concentrations of nicosulfuron, according to Duncan’s test. NS: nicosulfuron; IE: isoxadifen-ethyl.

**Fig 6:**
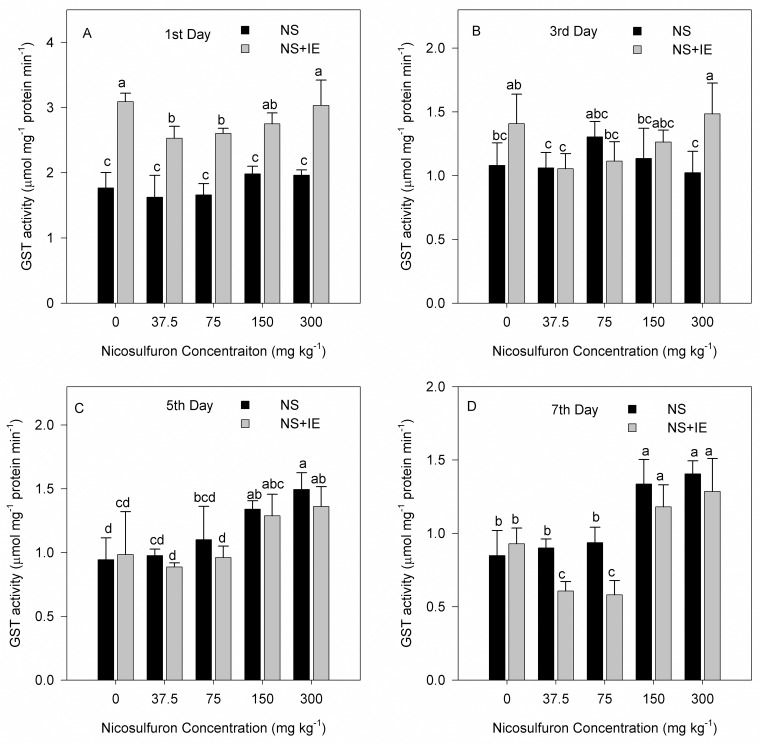
Changes in GST activity in maize Zhenghuangnuo No. 2 exposed to nicosulfuron alone or in combination with isoxadifen-ethyl after treatment. The different lowercase letters are significantly different from each other (P < 0.05) among different concentration of nicosulfuron, according to Duncan’s test. NS: nicosulfuron; IE: isoxadifen-ethyl.

For maize cultivar Zhengdan 958, the highest GST activity was found at 2.73 μmol min^-1^ mg^-1^ protein on the third day of treatment with isoxadifen-ethyl alone (Fig 5). For Zhenghuangnuo No.2 cultivar, the highest GST activity was found at 3.09 μmol min^-1^ mg^-1^ protein on the first day of treatment with isoxadifen-ethyl alone (Fig 6). In addition, GST activity of the two maize hybrids showed enhancement in the treatment with isoxadifen-ethyl relative to the control at all points in time. The GST activity in the two varieties treated with isoxadifen-ethyl safener plus nicosulfuron on the first and third days increased relative to the nicosulfuron only treatment, whereas the activity on the fifth and seventh days decreased. In each case, maximal induction of maize Zhengdan 958 was seen on the third day, whereas Zhenghuangnuo No. 2 was on the first.

### GST induction studies with isoxadifen-ethyl

**Fig 7:**
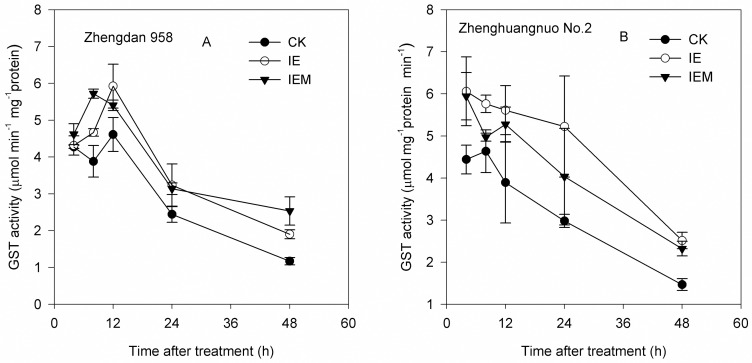
The effect on GST activity over 48 h after treatment of 7-day-old maize shoots with 33 mg kg^-1^ isoxadifen-ethyl, IEM, and control. CK: non-treated control; IE: isoxadifen-ethyl; IEM: 4,5-dihydro-5,5-diphenyl-1,2-oxazole-3-carboxylic. All treatments were applied with a non-ionic surfactant (0.1% v/v).

To evaluate the dynamics of GST induction in greater detail, untreated seven-day-old seedlings were treated with 33 mg kg^-1^ isoxadifen-ethyl. The induction of GST activity was then determined over a 48 h period (Fig 7). GST activity of maize cultivar Zhengdan 958 was significantly enhanced relative to control within 12 h of treatment, while in maize cultivar Zhenghuangnuo No. 2, GST activity was significantly enhanced relative to control within 4 h of treatment, with no further increase in GST activity determined over the remaining period up to 48 h. This showed that GST induction had occurred very quickly following exposure to the safener. In addition, GST induction in maize Zhenghuangnuo No. 2 was faster than in Zhengdan 958. Taylor et al. [34] showed that isoxadifen-ethyl was absorbed through the foliage and de-esterified a free acid, which is thought to be the active agent. To determine if isoxadifen acid was active as a safener, the parent ester was hydrolyzed to yield the free acid, which was then used to treat the maize shoots, with the induction of GSTs again monitored over a 48 h period (Fig 7). The results showed that the free acid gave an essentially identical fold-induction to the parent ester in both maize hybrids. These results suggested that isoxadifen acid is in fact the active safener, with the ester effectively acting as an uncharged and hydrophobic precursor that assists in the rapid delivery of the compound across the waxy cuticle of the maize leaf. As such, the effective bioactivation of the safener is analogous to the uptake, hydrolysis, and activation of herbicide eaters [35].

## Discussion

While herbicides contribute to improving crop yield, they can also pose a risk to plants that are sensitive to them [36]. The use of herbicide has currently resulted in an increased incidence of herbicidal injury [37,38]. For that reason, it has become essential to develop effective safeners. In order to develop a safener for nicosulfuron, the protective effects of five safeners were evaluated in our laboratory. The results conclusively demonstrated that maize plants injured by nicosulfuron were effectively protected by isoxadifen-ethyl.

Under greenhouse conditions, nicosulfuron reduced shoot length and shoot dry weight of two maize hybrids. Shoot length and shoot dry weight of maize decreased with the increase in nicosulfuron concentrations. The combination of nicosulfuron and PBO, ABT, or malathion caused significant injury to maize seedlings. Maize injury from nicosulfuron applied at 37.5–300 mg kg^-1^ was reversed by the safener isoxadifen-ethyl. The increased maize injury from PBO, ABT, or malathion plus nicosulfuron at 20 mg kg^-1^ was reversed by isoxadifen-ethyl. The enhanced tolerance of maize to nicosulfuron in the presence of safener, coupled with the enhanced injury observed in the presence of PBO, ABT, and malathion, suggested P450 may be involved in metabolism of nicosulfuron. Previous research has found that cloquintocet-mexyl antagonized the effects of malathion or ABT on topramezone, but the implications of this are not clear. It is possible that cloquintocet-mexyl increased topramezone metabolism through mechanisms not catalyzed by P450 monooxygenases [39,40]. We speculated that isoxadifen-ethyl increased plant metabolism of nicosulfuron through non-P450-catalyzed routes or through P450 monooxygenases not inhibited by PBO, ABT, and malathion. Yun et al. [40] evaluated the effects of the P450 monoxygenase inhibitor PBO alone and in combination with safeners on the activity of microsomal pyrazosulfuron-ethyl O-demethylase, which metabolizes pyrazosulfuron-ethyl in rice (*Oryza sativa* L.). They found that PBO inhibited 63% of pyrazosulfuron-ethyl O-demethylase activity when applied in combination with the herbicide, but only 19% inhibition was achieved when PBO was applied in combination with the herbicide and the safener. The researchers suggested that this was because the herbicide and safeners induce different P450 monooxygenases. Previous research indicates that ABT inhibits P450 monooxygenases responsible for aryl ring hydroxylation but not those responsible for N-demethylation [40].

The use of a herbicide safener could increase crop tolerance by inducing the activity of detoxifying enzymes, such as P450s, GSTs, or other detoxifying enzymes such as glucosyltransferases or ABC transporters [20, 41]. For evaluation of the enhancement of detoxification of maize, induced by safeners, the GSH content, GST activity, and ALS activity of maize treated by nicosulfuron and safener isoxadifen-ethyl were studied. Our results indicated that the isoxadifen-ethyl safener applied alone and premixed with nicosulfuron was not responsible for inducing GSH content. The safener isoxadifen-ethyl caused enhancement of GST activity, which is in agreement with previous studies [19, 31]. According to Matola and Jablonkai [35], the protective efficacy of dichloroketal safeners in maize does not seem to be associated with enhanced herbicide detoxification by glutathione conjugation. Several studies have reported that safeners increase herbicide tolerance given that they selectively induce GST activity [42,43]. It should also be considered that GSTs include constitutive isozymes as well as safener-inducible isozymes [34]. In the studies of isoxadifen-ethyl, we were unable to identify any downstream metabolites other than the free acid IEM. By demonstrating that IEM was as active at inducing GSTs as the parent ester, these studies demonstrated that the acid was more likely to be the direct source of safener activity. Consistent with this notion, it has been shown that isoxadifen-ethyl was absorbed through the foliage and de-esterified, resulting in a free acid that is thought to be the active agent [38].

Nicosulfuron inhibits ALS activity and which blocks branched-chain amino acid biosynthesis. So, ALS activity has a close relationship to plant resistance to nicosulfuron. The results suggested that the ALS activity of maize was improved by isoxadifen-ethyl. It was one of elementary pathways for herbicide detoxication in crops. Our results also showed that there was no difference in their herbicide sensitivity at the whole-plant level, but they differed in the sensitivity of ALS activity from these cultivars to nicosulfuron inhibition *in vitro* by 27-fold. We hypothesized that nicosulfuron resistance is based primarily on metabolic detoxification.

In summary, isoxadifen-ethyl provides protection against injury caused by nicosulfuron to maize cultivar Zhengdan 958 and waxy maize cultivar Zhuanghuangnuo No. 2. The inducement of GST activity was accompanied by the release of ALS activity in maize treated with nicosulfuron and isoxadifen-ethyl. This suggested that the detoxification process may be accompanied by the induction of the target enzyme. Further studies will focus on the expression of GST genes in maize seedlings exposed to isoxadifen-ethyl.
